# Neonatal Cerebral Sinovenous Thrombosis and the Main Perinatal Risk Factors—A Retrospective Unicentric Study

**DOI:** 10.3390/children9081182

**Published:** 2022-08-07

**Authors:** Catalina Filip, Gabriela Ildiko Zonda, Ingrid-Andrada Vasilache, Ioana Sadiye Scripcariu, Petronela Vicoveanu, Vlad Dima, Demetra Socolov, Luminita Paduraru

**Affiliations:** 1 Saint Spiridon University Hospital, Vascular Surgery Clinic, Independence Boulevard No. 1, 700111 Iasi, Romania; 2 Division of Neonatology, Department of Mother and Child Care, “Grigore T. Popa” University of Medicine and Pharmacy, 700111 Iasi, Romania; 3 Division of Obstetrics and Gynecology, Department of Mother and Child Care, “Grigore T. Popa” University of Medicine and Pharmacy, 700115 Iasi, Romania; 4 Department of Obstetrics, Gynecology and Neonatology, Filantropia Clinical Hospital, 11-13 Ion Mihalache Blv., Sector 1, 011171 Bucharest, Romania

**Keywords:** cerebral sinovenous thrombosis, neonatal, risk factors, inherited thrombophilia

## Abstract

(1) Background: Neonatal cerebral sinovenous thrombosis (CSVT) is a rare disorder, associated with long-term neurological sequelae. The aim of this study was to retrospectively evaluate the most commonly encountered perinatal risk factors for this disease in a cohort of newborns from Romania. (2) Methods: The medical records of neonatal CSVT patients treated between January 2017 and December 2021 were descriptively assessed. (3) Results: The study included nine neonates, five males (55.56%) and four females (44.44%), who were born at term. The most commonly presented clinical manifestations were feeding difficulties, lethargy, respiratory distress, loss of consciousness, and seizures. Maternal-inherited thrombophilia, male sex, complicated delivery, perinatal asphyxia, and mechanical ventilation were frequently identified as potential risk factors for developing CSVT. The lesions were more frequently localized in the superior sagittal sinus (n = 7; 77.78%), followed by the transverse (n = 4; 44.44%), sigmoid (n = 2; 22.22%), and cavernous (n = 1; 11.11%) sinuses. Low-molecular-weight heparin was administered to all patients, and two of them died from thrombotic complications. (4) Conclusions: Recognition of potential risk factors and a prompt diagnosis of neonatal CSVT could lead to better patient management and to a reduction of severe complications.

## 1. Introduction

A focal or widespread interruption of cerebral blood flow caused by the obstruction of cerebral veins and/or sinuses is known as cerebral sinovenous thrombosis (CSVT) [1]. This pathologic entity can manifest as a result of multiple etiologies and has a polymorphic clinical spectrum; thus, its diagnosis can be challenging. 

Although CVST is a rare condition, it appears that neonates are the most commonly affected pediatric age group, with an estimated combined incidence of approximately 1:1600–1:2300 live births [2,3,4]. The reported incidence of neonatal thrombosis over the last few years varied between 6.9–15 per 1000 neonates admitted to the intensive care unit (NICU) [5,6,7,8], and it appears to be higher than the previously reported incidence in the literature published in the 1990s and early 2000s (2.4–5.5 per 1000 admissions to the NICU) [9,10]. For cerebral sinovenous thrombosis, the Dutch registry reported an incidence ranging from 1–12 per 100.000 newborns, whereas the Canadian registry reports a higher rate of 47 per 100.000 newborns [2,11]. 

Several maternal, fetal, and neonatal risk factors for CSVT have been cited in the literature and include preeclampsia, gestational diabetes, inherited thrombophilia, complicated delivery, perinatal asphyxia, neonatal sepsis, prolonged arterial or venous catheterization, dehydration, and meningitis [12,13,14]. Maternal- and/or neonatal-inherited thrombophilia is an important procoagulant risk factor that could lead to the development of obstructive thrombi in the cerebral venous system, and specific thrombophilia mutations, such as a factor V Leiden homozygous mutation, prothrombin gene G20210A mutation, and methylenetetrahydrofolate reductase (MTHFR) 677TT mutation, and protein C, S, and antithrombin III deficiencies are recognized as having a significant effect on thrombotic manifestations [13,15,16]. Moreover, a combination of the previously mentioned risk factors increases the odds of neonatal CSVT. 

The clinical manifestations in neonates are often subtle and nonspecific, especially at the presentation, and include seizures, lethargy, irritability, poor feeding, or changes in muscle tone [17,18]. The CSVT-associated intracranial hypertension syndrome is a medical emergency, which can lead to brain tissue herniation and death [19]. 

The diagnosis of neonatal CSVT should be made as soon as possible, and imaging examinations are the main diagnostic tools. Thus, magnetic resonance imaging (MRI) and MRI venography can be used primarily to assess venous thrombus in terms of size and location, as well as to quantify brain parenchymal lesions [20]. When magnetic resonance imaging is not possible, the use of computed tomography (CT) and CT venography is recommended, which, although highly sensitive for the detection of venous thrombi, does not allow a complete description of brain parenchymal lesions [21]. 

Therapeutic strategies for newborns affected by neonatal cerebral venous thrombosis are based on treatment guidelines for adults with the same pathology as the condition is rare in the perinatal period. Therefore, supportive and anticoagulant treatments are the main therapeutic pillars in these cases [22]. 

Endovascular permeabilization techniques could also be evaluated as therapeutic options under experimental conditions, as evidenced by data from studies in small groups of newborns [23,24]. The prognosis of newborns affected by CSVT is variable. More than 90% of them survive an acute episode, but neurological sequelae have a wide range of manifestations [25]. 

Regarding the association between specific risk factors and neonatal cerebral venous thrombosis, the data in the literature are extremely limited, probably due to the rarity of the disease. In this study, we aimed to highlight the most commonly encountered clinical risk factors for neonatal CSVT in a cohort of neonatal patients from a tertiary center in Romania. 

## 2. Materials and Methods

In this observational, retrospective, unicentric study, we evaluated the neonatal CSVT cases admitted to a Level III neonatal intensive care unit from the Clinical Hospital of Obstetrics and Gynecology “Cuza-Voda”, Iasi, Romania, between January 2017 and December 2021. Ethical approval for this study was obtained from the Institutional Ethics Committee of the regional hospital (No. 9272/19 July 2022). Informed consent was obtained from all participants included in the study. All methods were carried out in accordance with relevant guidelines and regulations. 

The inclusion criteria comprised all cases with CSVT diagnosed in the neonatal period from our tertiary care center, whereas the exclusion criteria included incomplete medical records or the mother’s inability to provide informed consent. 

Medical records of 9 neonates with cerebral sinovenous thrombosis were systematically reviewed and data were obtained. The patients’ clinical characteristics (gestational age, birthweight, Apgar score at 1 and 5 min, length and head circumference), risk factors for CSVT, and clinical manifestations and outcomes, as well as the diagnostic and therapeutic approaches taken, were documented. 

Confirmation of the clinical diagnosis was made on the basis of a complete neurological clinical examination and magnetic resonance imaging performed at the Pediatric Emergency Clinical Hospital “Saint Mary” from Iasi, based on a collaboration agreement. Data were collected on the onset and evolution of neonatal neurological symptoms, imaging location of the thrombus, and imaging aspect of the condition.

Data were analyzed with the SPSS 17.0 statistical software package (SPSS Inc., Chicago, NY, USA) using descriptive statistics.

## 3. Results

The medical data from nine neonates was taken into consideration for description, and their clinical characteristics are presented in Table 1. Individual case-level details are presented as supplementary data (Table S1). The mean gestational age at birth and standard deviation (SD) was 39.1 ± 1.26 weeks of gestation. The mean birthweight and SD were 3493.3 ± 645.9 g. The mean Apgar score at 1 min and SD were 4.2 ± 2.4, ranging from 1 to 7, whereas the mean Apgar score at 5 min and SD were 6 ± 2.3, ranging from 2–8. 

Mothers of seven neonates (77.78%) were diagnosed with inherited thrombophilia (Table 2), and specific thrombophilic mutations were detected in the case of two patients: one was positive for the heterozygous mutation of factor V Leiden and methylenetetrahydrofolate reductase (MTHFR) C667T, whereas the other presented the heterozygous mutation of factor V Leiden (4070 A > G), heterozygous genotype 4G/5G of the plasminogen activator inhibitor-1 (PAI-1) gene, heterozygous genotype T1565C of the glycoprotein IIIa, heterozygous genotype of the angiotensin-converting enzyme (ACE), e3/e4 mutation of the apolipoprotein E, heterozygous genotype Met/Thr of the angiotensinogen (AGT) gene, heterozygous genotype of the Angiotensin II type 1 receptor (ATR-1), and heterozygous genotype 455 G > A of the fibrinogen gene. 

The majority of the patients were males (n = 5; 55.56%), and were born through vaginal delivery (n = 6; 66.67%). A complicated delivery was determined in four cases of perinatal asphyxia (44.44%) and in one case of shoulder dystocia (11.11%). Meconium aspiration and respiratory distress associated with delivery complications manifested in four cases (44.44%). Mechanical ventilation was required in eight cases (88.89%), either immediately after birth or after the onset of severe clinical manifestations such as tonic–clonic seizures. 

The mean time interval and SD until the onset of clinical manifestation were 3.22 ± 0.64 days (95% CI: 1.74–4.69), and the main symptoms were represented by feeding difficulties (n = 8; 88.89%), lethargy (n = 7; 77.78%), respiratory failure (n = 4; 44.44%), and loss of consciousness and seizures (n = 3; 33.33%), and only one case (11.11%) presented with bilateral inferior limb spasticity (Table 3).

In three cases affected by tonic–clonic seizures, an electroencephalogram was performed, and it revealed spike and wave discharges. Diagnosis of neonatal CSVT was confirmed in all neonates using a 1.5 Tesla MRI examination, and differential diagnosis included ischemic arterial stroke or possible congenital cerebral malformation. Multiple sinuses were affected by thrombosis in four cases (44.44%), and the most frequent localization of the lesion was the superior sagittal sinus (n = 7; 77.78%), followed by transverse (n = 4; 44.44%), sigmoid (n = 2; 22.22%), and cavernous (n = 1; 11.11%) sinuses (Table 4). Associated cerebral lesions included diffuse cerebral edema (n = 3; 33.33%), intraventricular hemorrhage (n = 2; 22.22%), thalamic hemorrhage, choroid plexus hemorrhage, and ventriculomegaly (n = 1; 11.11%). 

All patients received supportive and anticoagulant treatment. Low-molecular-weight heparin (LMWH) was preferred to unfractionated heparin due to its ease of administration, particularly in newborns. All neonates received anticoagulant therapy of LMWH (Fraxiparin 100 units/day) for 3 months. None of these neonates had intracranial hemorrhage subsequent to anticoagulation. Two infants (22.22%) died due to their thrombotic complications in the first year of life. One of them developed an intracardiac thrombus that led to a cerebral stroke, whereas the other developed acute mesenteric ischemia due to a thrombus. 

## 4. Discussion

Perinatal cerebral venous thrombosis is a rare condition associated with significant morbidity and mortality in newborns. Although the interval of manifestation of this disease is cited as being between 28 weeks of gestation and 28 days postpartum, most cases (81%) occur in the first week of life [17]. In this study, clinical manifestations were detected in the first week of life.

As in any other thrombotic process, risk factors are associated with the components of the Virchow triad: hypercoagulability, vascular injury, and vascular stasis. Regarding the contribution of mutations specific to hereditary thrombophilia to the occurrence of perinatal cerebral venous thrombosis, data from the literature are limited to several studies.

In a retrospective study evaluating the clinical manifestations, treatment, and outcomes of 42 neonates with neonatal cerebral venous thrombosis, the authors found that 3 patients (13%) out of 24 tested had heterozygous mutations of factor V Leiden, and 4 (40%) out of 10 tested had the C677T mutation of the MTHFR gene, whereas 4 (44%) out of 9 tested had the A1298C mutation of the MTHFR gene [17].

Heller et al., compared prothrombotic risk factors in 149 children with cerebral venous thrombosis and 149 healthy children in the control group, and identified a statistically significant association between the condition studied and mutations in factor V Leiden, protein C, and S genes [26].

A meta-analysis performed by Laugesaar and colleagues analyzed the association between pediatric CSVT and mutations of factor V Leiden and prothrombin, respectively. The results indicated a statistically significant association between the G20210A prothrombin gene mutation (OR: 3.1; 95% CI: 1.4–6.8) and the Leiden factor V mutation (OR: 3.1; 95% CI: 1.8–5.5) and cerebral venous thrombosis [27].

The peculiarity of two presented cases consists of the occurrence of neonatal cerebral venous thrombosis in newborns with hereditary thrombophilia, whose mothers were also diagnosed with hereditary thrombophilia. Mutations of factor V Leiden, MTHFR, and PAI-1 were the polymorphisms with the most important thrombogenic potential identified in the analyzed cases. Due to increased costs, many families could not afford the thrombophilia screening tests for their infants. This is the reason why only two of the evaluated patients had thrombophilia panels. On the other hand, mothers of seven of the children were diagnosed with inherited thrombophilia during evaluation in our tertiary center that focuses on the management of high-risk pregnancies. 

For PAI-1 there was no data in the literature confirming the significant association with perinatal cerebral venous thrombosis, whereas the MTHFR mutations have been confirmed as risk factors for the studied pathology. Thus, a meta-analysis performed by Kenet et al. reported an increased risk for developing perinatal cerebral venous thrombosis in patients with mutations in MTHFR (OR: 1.58; 95% CI, 1.20–0.08) or factor V Leiden (OR: 3.26; 95% Cl, 2.59–4.10) [13].

Besides thrombophilia mutations, other maternal and neonatal risk factors have been cited as important contributors to the development of cerebral venous thrombosis. The univariate analysis of a nested case–control study revealed that male sex, preterm birth, hypoxia, and related indicators (umbilical artery pH < 7.1; 5 min Apgar score < 7; intubation/mask ventilation; perinatal asphyxia) were significantly associated with neonatal cerebral sinovenous thrombosis [18]. The majority of our patients were males, born through vaginal delivery, and had a mean Apgar score at 5 min and SD of 6 ± 2.3 (range: 2–8; 95% CI: 4.19–7.80). However, all of our patients were born at term. 

A complicated delivery (five cases), meconium aspiration (four cases) and respiratory distress (four cases) were identified as frequent risk factors to the development of neonatal cerebral venous thrombosis in our cohort of patients. Moreover, mechanical ventilation was required in eight cases (88.89%) either immediately after birth, or after the onset of severe clinical manifestations such as tonic–clonic seizures. 

It was outlined that in the immediate perinatal period, 60% of CSVT cases are attributable to a complicated delivery and perinatal asphyxia, whereas in the neonatal period, CSVT is often associated with systemic infection, meningitis, and dehydration [1]. Although we did not diagnose systemic infections or meningitis in our patients, the majority of them (n = 8; 88.89%) suffered from feeding difficulties, which could contribute to the development of cerebral venous thrombosis. 

Regarding the clinical manifestations, our patients had nonspecific symptoms such as lethargy or feeding difficulties with subacute onset, as well as neurological manifestations such as tonic–clonic seizures or spasticity. If in adults the clinical manifestations specific to an ischemic or hemorrhagic stroke are relatively easy to spot, in newborns it is important to follow the symptoms closely over time. The most common clinical manifestations of perinatal venous thrombosis cited in the literature are hypotonia, lethargy, eating or respiratory disorders, convulsive or nonconvulsive seizures, and changes in eyeball movements [2,28].

The most frequent localization of the lesions was the superior sagittal sinus (n = 7; 77.78%), followed by the transverse (n = 4; 44.44%), sigmoid (n = 2; 22.22%), and cavernous (n = 1; 11.11%) sinuses. Moreover, multiple sinuses were affected by thrombosis in four cases (44.44%). Our data were comparable to that published by the International Study on Cerebral Vein and Dural Sinus Thrombosis (ISCVT), which determined the frequency of the sites of sinovenous cerebral thrombosis as follows: transverse sinus, 86%; superior sagittal sinus, 62%; straight sinus, 18%; cortical veins, 17%; jugular veins, 12%; and vein of Galen and internal brain veins, 11% [29]. 

It was shown that full-term infants frequently present with punctate white matter lesions and intraventricular or thalamic hemorrhage, whereas preterm infants show extensive white matter lesions [30]. In our cohort of patients, who were all born at term, the most commonly associated cerebral lesions included diffuse cerebral edema (n = 3; 33.33%), intraventricular hemorrhage (n = 2; 22.22%), thalamic hemorrhage, choroid plexus hemorrhage, and ventriculomegaly (n = 1; 11.11%). 

It is debatable whether anticoagulation and antiplatelet therapy should be used to treat neonatal CSVT. A lack of randomized controlled studies and a high incidence of spontaneous intracranial and intraventricular hemorrhage in newborns raise concerns over their use [31]. On the other hand, the American College of Chest Physicians recommends the anticoagulant therapy with either low-molecular-weight or unfractionated heparin for a period of six weeks to three months for neonates diagnosed with CSVT [22]. 

A recent meta-analysis that evaluated whether anticoagulation therapy in the treatment of neonatal CSVT improves outcomes, in the presence or absence of a pre-existing intracerebral hemorrhage, demonstrated a reduced risk of propagation of thrombus (risk ratio 0.14, 95% CI: 0.03–0.72) with anticoagulant therapy, but failed to prove a significant effect on mortality before discharge either in the presence or absence of a pre-existing intracerebral hemorrhage of such treatment [32]. 

In our cohort of patients, all neonates received anticoagulant therapy of LMWH for 3 months without important adverse effects, but two of them succumbed from thrombotic complications. 

The present study has some limitations due to its retrospective and unicentric design, the small group of patients, and the limited number of variables taken into consideration for descriptive analysis. The main strength of this study is the descriptive presentation of an extremely rare condition in the neonatal period that has a complex clinical presentation and needs individualized management.

Further studies on larger cohorts of patients will be needed to evaluate the association between individual risk factors and cerebral venous thrombosis in the neonatal period.

## Figures and Tables

**Table 1:** Clinical characteristics of newborns with CSVT.

Clinical Characteristics	Mean ± Standard Deviation	Range	95% Confidence Interval
Gestational age, weeks	39.1 ± 1.26	37–41	38.13–40.08
Birth weight, g	3493.3 ± 645.9	2700–4700	2996.78–3989.88
Apgar score, 1 min	4.2 ± 2.4	1–7	2.34–6.09
Apgar score, 5 min	6 ± 2.3	2–8	4.19–7.80
Length, cm	50.8 ± 2	48–54	49.33–52.44
Head circumference, cm	35.3 ± 1	34–37	34.56–36.10

**Table 2:** The main risk factors for CSVT identified in our cohort of patients.

Risk Factor	N (%)
Sex	Male = 5 (55.56%) Female = 4 (44.44%)
Maternal thrombophilia	7 (77.78%)
Type of birth	Vaginal = 6 (66.67%) Cesarean = 3 (33.33%)
Meconium aspiration	4 (44.44%)
Small for gestational age	2 (22.22%)
Perinatal asphyxia	4 (44.44%)
Shoulder dystocia	1 (11.11%)
Nuchal chord	2 (22.22%)
Mechanical ventilation	8 (88.89%)
Respiratory distress	4 (44.44%)

**Table 3:** Clinical manifestations in newborns with CSVT.

Clinical Manifestations	N (%)
Lethargy	7 (77.78%)
Feeding difficulties	8 (88.89%)
Seizures	3 (33.33%)
Bilateral inferior limb spasticity	1 (11.11%)
Respiratory failure	4 (44.44%)
Loss of consciousness	3 (33.33%)

**Table 4:** Sinus involvement and associated cerebral lesions identified in our cohort of patients.

Type of CSVT	N (%)
Number of sinuses involved	Single sinus = 5 (55.56%) Multiple sinuses = 4 (44.44%)
Superior sagittal sinus	7 (77.78%)
Transverse sinus	4 (44.44%)
Sigmoid sinus	2 (22.22%)
Cavernous sinus	1 (11.11%)
Associated lesions	Diffuse cerebral edema = 3 (33.33%) Intraventricular hemorrhage = 2 (22.22%) Thalamic hemorrhage = 1 (11.11%) Choroid plexus hemorrhage = 1 (11.11%) Ventriculomegaly = 1 (11.11%)

## Data Availability

The data presented in this study are available on request from the corresponding author. The data are not publicly available due to local policies.

## Supplementary Materials

The following supporting information can be downloaded at: https://www.mdpi.com/article/10.3390/children9081182/s1, An extended table with individual case-level details can be accessed as Table S1: Clinical and imagistic findings in a cohort of newborns with CSVT.
