# Supplementary material for: Neonatal Cerebral Sinovenous Thrombosis and the Main Perinatal Risk Factors—A Retrospective Unicentric Study
Source: Children (Basel). 2022 Aug 7;9(8):1182. doi: 10.3390/children9081182 (PMC9406898; doi:10.3390/children9081182)
Supplement: Supplementary file 1 [file children-09-01182-s001.zip › children-1848188-supplementary.pdf]

Table S1. Clinical and imaging findings in a cohort of newborns with CSVT.

Patient's Characteristics		Patient's Number								
		**1**	**2**	**3**	**4**	**5**	**6**	**7**	**8**	**9**
**Clinical findings**	Gestational age, weeks	38	41	38	40	40	39	40	37	39
	Birth weight, g	4700	3100	2970	3350	3750	2700	4020	2950	3900
	Apgar score 1 minute	3	1	7	5	1	4	3	7	7
	Apgar score 5 minutes	5	2	8	7	3	8	5	8	8
	Length, cm	53	50	50	49	54	50	53	48	51
	Head circumference, cm	37	35	34	35	36	35	36	34	36
	Gender	M	F	F	M	M	F	M	F	M
	Type of birth	Vaginal	Vaginal	Cesarean	Vaginal	Cesarean	Vaginal	Vaginal	Vaginal	Cesarean
	Maternal thrombophilia	Yes	Yes	Yes	Yes	Yes	Yes	Yes	No	No
	Meconium aspiration	No	Yes	Yes	No	Yes	No	No	Yes	No
	Perinatal asphyxia	Yes	Yes	No	No	Yes	Yes	No	No	No
	Shoulder dystocia	Yes	No	No	No	No	No	No	No	No
	Nuchal chord	No	No	No	Yes	Yes	No	No	No	No
	Mechanical Ventilation	Yes	Yes	Yes	No	Yes	Yes	Yes	Yes	Yes
**Symptoms**	Respiratory distress	Yes	No	Yes	No	No	Yes	No	No	Yes
	Lethargy	Yes	Yes	Yes	No	Yes	Yes	Yes	No	Yes
	Feeding difficulties	Yes	Yes	Yes	Yes	Yes	No	Yes	Yes	Yes
	Seizures	No	Yes	No	No	No	Yes	No	Yes	No
	Bilateral inferior limb spasticity	No	No	No	No	No	No	No	Yes	No

	Loss of consciousness	No	Yes	No	No	No	Yes	No	Yes	No
	Superior sagittal sinus	Yes	No	Yes	No	Yes	Yes	Yes	Yes	Yes
	Transverse sinus	No	Yes	No	No	No	Yes	Yes	No	Yes
	Sigmoid sinus	No	No	No	Yes	No	No	No	No	Yes
**Imaging findings**	Cavernous sinus	No	No	No	No	No	No	No	No	Yes
	Associated lesions	Intraventricular hemorrhage Thalamic hemorrhage	Diffuse cerebral oedema Choroid plexus hemorrhage	No	Diffuse cerebral oedema	Diffuse cerebral oedema	Intraventricular hemorrhage	Ventriculomegaly	No	No
Survival al 1 year		Alive	Alive	Dead	Alive	Alive	Alive	Dead	Alive	Alive
